# Conformational and Functional Properties of Soybean Proteins Produced by Extrusion-Hydrolysis Approach

**DOI:** 10.1155/2018/9182508

**Published:** 2018-05-23

**Authors:** Wenjun Ma, Baokun Qi, Rokayya Sami, Lianzhou Jiang, Yang Li, Hui Wang

**Affiliations:** ^1^College of Food Science, Northeast Agricultural University, Harbin 150030, China; ^2^Department of Food Science and Nutrition, Taif University, Taif, Al-huwayah 888, Saudi Arabia; ^3^Harbin Institute of Food Industry, Harbin 150030, China; ^4^Center for Crops Utilization Research, Iowa State University, Ames, IA, USA

## Abstract

The conformational and functional changes of soybean protein after a hybrid extrusion-hydrolysis method were evaluated. Three extrusion temperatures (60, 80, and 100°C) were used prior to enzymatic hydrolysis. The hydrolysis degrees, molecular weight profiles, solubilities, surface hydrophobicities, sulphydryl contents, disulfide bound, water holding capacity, emulsion, and foam properties of the protein isolated from the enzyme-hydrolyzed extruded soybeans were analyzed. It shows that extrusion caused significant changes in the hydrophobicity, molecular weight distribution, solubility, surface hydrophobicity, emulsification activity, and stability of the protein. The increase of molecular weights could be attributed to the formation of protein aggregates during extrusion. Extrusion and enzymatic hydrolysis led to a sharp increase in the number of disulfide bonds with a decrease of the sulphydryl group. The water holding capacity and the solubility of protein increased with the increase of extrusion temperature and hydrolysis time. Extrusion improved the emulsifying activity but reduced the emulsifying stability of the recovered proteins. Extrusion improved the foam capacity but reduced the foam stability of the proteins. The data demonstrated that the extrusion-hydrolysis treatment significantly altered the conformational and functional properties of soybean protein, which may be further optimized for the development of new soy protein ingredient with desired functional properties.

## 1. Introduction

Soybean is one of the most important oilseed kinds in the world. It is source of vegetable protein with high nutrition values [1] and versatile applications in food products at a reasonable cost [2]. The uses of soybean protein in food or nutraceutical products has been increasing steadily due to the health claim authorized by the Food and Drug Administration (FDA) in 1999 that “25 grams of soy protein a day, as part of a diet low in saturated fat and cholesterol, may reduce the risk of heart disease.”

Extrusion technique has been applied for soy foods processing recently, which is well known as a texturized soy protein-based meat analog [3, 4]. Extrusion can result in the disruption of compact quaternary and tertiary structures of soy protein and also can improve the efficiency of enzymatic hydrolysis [5]. Therefore, enzymatic hydrolysis is often applied after extrusion to modify the functional properties of soy protein. Clemente [6] reported that enzymatic hydrolysis can enhance or reduce the functional properties and nutritional value of soy protein. Chen et al. [7] found that extrusion increased the accessibility of soy protein to enzymatic hydrolysis and improved the emulsification properties of the protein. Lamsal et al. [8] evaluated the functional properties of the enzyme-hydrolyzed extruded soybean flour and demonstrated the extrusion process which affected the functional and sensory properties of soy protein. Furthermore, Surowka et al. [9] found that after extrusion, all the subunits of soybean protein were rapidly degraded by enzyme. Although there is abundant evidence that the extrusion and associated enzymatic hydrolysis could alter the structural and functional properties of soy protein, most of the previous extrusion studies used soy protein isolate power. There is a rare study in that field, dealing with the behaviors of soy protein isolated from extruded whole soybeans at different extrusion temperature, as well as the impact of consequent enzymatic hydrolysis on the extruded soy protein.

This study was designed to (1) investigate the effects of extrusion temperatures and associated enzymatic hydrolysis on the conformational changes of soy protein and (2) quantify the conformational and functional properties of the protein.

## 2. Materials and Methods

### 2.1. Materials

Full-fat soybean flakes (from soybean cultivar Dong-Nong 42, harvested in 2017 in Harbin, China) containing 20% fat and 40% crude protein on a dry basis were obtained from Lanshan Group (Shandong, China). The flakes were sealed and stored in plastic bags at 4°C until further use. Protex 6L (alkaline serine endopeptidase from* Bacillus licheniformis*, 580000 DU/g) was purchased from Novozymes (Tianjin, China). Phthaldialdehyde (OPA), 5,5′-dithio-bis 2-nitrobenzoic acid (DTNB), 2-nitro-5-thiosulphobenzoate (NTSB) and 1-anilino-8-naphthalenesulphonate (ANS) were purchased from Sigma-Aldrich (St. Louis, MO, USA). All other reagents were of analytical grade.

### 2.2. Extrusion Treatment

A corotating twin screw extruder (Evolum 25, Clextral, Firminy, France) equipped with a feeder (model T20, K-TRON (Schweiz) AG, Hillenbrand, France) was used in all extrusion treatments. The screw has a length to diameter ratio of 32 : 1 and 11 sections of conveying and kneading screws. The extrusion conditions were as follows: feed rate: 50 g/min; 5-barrel temperatures sections were 30, 30, 40, 40, and 50°C. Preliminary extrusion runs showed that full-fat soy flakes needed to contain 16% moisture for satisfactory extrusion. The moisture content of the extruder during extrusion was adjusted to 16% by adding water to full-fat soybean flakes directly. The soybean flakes were first ground into powder using a laboratory grinder (FW-100, Shaoxing Kehong Instrument Co., Ltd., Zhejiang, China) before passing through a 60-mesh sieve (<250 *μ*m). The only change was the die temperatures, which was set at 60, 80, and 100°C, respectively, for each treatment. The extruder was cooled to room temperature before grinding into fine powder (<250 *μ*m). The final soybean powder was stored in plastic bags at 4°C until subsequent enzymatic hydrolysis treatments.

### 2.3. Enzymatic Hydrolysis Treatment

Soybean flakes with and without extrusion were ground and subjected to pass through a 60-mesh sieve [10]. Afterwards, the flour was added to water to achieve solids-to-water ratio of 1 : 6 (w/v). The pH of the slurry was adjusted to 9 through the addition of 2 N NaOH. The slurry was incubated at 50°C in a water bath. Protex 6L was added to the slurry at a dosage of 1.85% (v/w, based on the dry weight of the soybean flour). A continuous stirring device was used to disperse the slurry during the incubation period. The hydrolysis duration was set to 0.5, 1.0, 1.5, 2.0, 2.5, and 3.0 h, respectively. Enzyme was then thermally deactivated. Finally, the slurry was centrifuged at 8,000 ×g for 20 min at 20°C (model TGL-16G, Anting Scientific Instrument Factory, Shanghai, China). The aqueous phase was carefully collected for subsequent isolation of protein.

### 2.4. Protein Isolation

The isolation of protein from the aqueous phase was initiated by adjusting the pH of the aqueous phase to 4.5 using 0.2 M HCl, in order to precipitate out the protein. The protein was subsequently collected by centrifugation at 10,000 ×g, 4°C for 15 min. The acid-precipitated protein was washed four times and neutralized using NaOH (2.0 M). Afterwards, the protein-rich solution was subjected to freeze-drying in a freeze dryer (FEtseries, GOLD SIM, Newark, USA) to isolate the hydrolyzed soybean protein powder (HSPP). The crude protein of the HSPP ranged from 70% to 78%, Nitrogen contents were converted to crude protein contents using a factor of 6.25.

### 2.5. Hydrolysis Degree Determination

The degree of hydrolysis (DH) is defined as the ratio of the number of peptide bonds hydrolyzed to the total number of peptide bonds per unit weight present in HSPP. The DH of each HSPP sample was determined using the modified OPA method [11]. The method was modified by adjusting 1.5 mL OPA reagents to 200 *μ*L of standard, blank andHSPP sample in individual tubes then left to react for 2 min. The absorbance was recorded at 340 nm (Lengguang Technology Co., Ltd., Shanghai, China). The DH% was calculated using

(1) Serine  NH2=ODsample−ODblankODstandard−ODblank×0.9516 meqv/L×0.1×100X×P L/gH=Serine  NH2−βαDH=hhtot×100,

where *h* = number of hydrolyzed bonds; *h*_tot_ = total number of peptide bonds per protein equivalent (7.8 specific to soybean protein); *β* = 0.342 (specific for soybean protein); *α* = 0.970 (specific for soybean protein); OD_sample_ = absorbance of sample at 340 nm; OD_blank_ = absorbance of water at 340 nm; OD_standard_ = absorbance of l-serine at 340 nm; *X* = amount of sample (g); and *P* = protein (%) in sample.

### 2.6. SDS-PAGE Analysis

Sodium dodecyl sulfate-polyacrylamide gel electrophoresis (SDS-PAGE) was carried out using the discontinuous system (15% separating/4% stacking gel) [12, 13]. Aliquots of each sample were mixed with 2x sample dissolving buffer (4% SDS, 20% glycerol, 0.125 M Tris-HCl buffer pH 6.8, 0.02% bromophenol blue). SDS-PAGE was carried out under reducing and nonreducing conditions. For reducing SDS-PAGE, 80 *μ*L of sample (2 mg/mL) was mixed with 20 *μ*L of 10% SDS, 2 *μ*L of 2-mercaptoethanol mercaptoethanol and 1 *μ*L of 1% w/v bromophenol blue. For nonreducing SDS-PAGE, 2-mercaptoethanol was not added. The samples were heated at 100°C for 5 min and centrifuged at 15,000 ×g for 10 min at room temperature. The supernatant was collected and loaded onto SDS-PAGE gels. The electrophoresis experiments ran at 80 V and subsequently ran at 120 V until the dye reached the bottom of the gel. After electrophoresis, the gel was stained with 0.05% (w/v) Coomassie blue R250 in 15% (v/v) methanol and 5% (v/v) acetic acid and destained with 30% (v/v) methanol and 10% (v/v) acetic acid. High molecular weight markers (Solarbio Co., Ltd., Beijing, China) were used to estimate the molecular weight of proteins.

### 2.7. High-Performance Size Exclusion Chromatogram

High-performance size exclusion chromatography (HPSEC) was carried out according to the method [14]. All samples were dissolved in water to a concentration of 1 mg/mL and then centrifuged at 10,000 ×g (25°C) for 10 min. The supernatant was filtered through a cellulose acetate membrane with a pore size of 0.22 *μ*m. The resulting solution was injected into the ÄKTA purifier system equipped with a Superdex™ 75 10/300 GL. The mobile phase consisted of 50 mM of phosphate buffer and 300 mM of NaCl. Data collection was performed at 214 nm using Unicorn Software (Version 5.01). Peak molecular weight ranges were estimated based on the calibration curve from a series of protein standards (thyroglobulin, bovine–globulin, chicken ovalbumin, equine myoglobin and vitamin B_12_).

### 2.8. Sulphydryl and Disulphide Bond Contents

HSPP at 15 mg were dissolved in 5 mL pH 8.0 buffer (0.086 M Tris, 0.09 M glycine, and 0.04 M Na_2_EDTA) with (total sulphydryl) or without 8 M urea (exposed sulphydryl) [15]. After that, 50 *μ*L of Ellman's reagent (4 mg DTNB/mL buffer) were added. The resultant slurry was incubated at 25°C for 1 h, prior to centrifugation at 5000 ×g for 15 min (model TGL-16G, Anting Scientific Instrument Factory, Shanghai, China). The absorbance of the supernatant was measured at 412 nm (SHJH Co. Ltd., Shanghai, China) using the Tris–Gly buffer containing Ellman's reagent as the blank. Sulphydryl groups were determined using

(2) $$\begin{array}{c} \mu mol\;\;SH/g=\frac{73.53\times A_{412}\times D}{C}, \end{array}$$

where *A*_412_ is the absorbance at 412 nm, *D* is the dilution factor, *C* is the sample concentration (mg/mL), and.53 is derived from 10^6^/(1.36 × 10^4^), and 1.36 × 10^4^ is the molar absorptivity.

Freshly prepared NTSB assay solution (3 mL) was added to 2 mL of HSPP solution, and then the mixture was incubated in the dark at room temperature for 25 min. After the reaction, the absorbance of the solution was measured at 412 nm using the test NTSB solution as the reference. The total disulphide content was calculated using a molar extinction coefficient of 13,900 M^−1^ cm^−1^ [16].

### 2.9. Solubility Measurement

To determine protein solubility, 200 mg of HSPP was dispersed in 20 mL water. The solution was centrifuged at 10,000 ×g for 10 min. After an appropriate dilution, the nitrogen content of the supernatant was determined by the Kjeldahl method and the solubility was expressed as g soluble nitrogen/100 g nitrogen in the sample (% NS).

### 2.10. Surface Hydrophobicity Measurement

HSPP samples were dissolved in a pH 7.6 phosphate buffer at 1 mg/mL and centrifuged at 8,000 ×g at 4°C for 20 min [17]. An aliquots of 10 mL supernatant was mixed with 50 mL of ANS (8.0 mmol/L in 0.05 mol/L phosphate buffer pH 7.6). Fluorescence intensity (FI) was measured at 330 nm (excitation) and 490 nm (emission) using an F-4500 spectrometer (Hitachi, Ltd., Tokyo, Japan) with excitation and emission slit of 5 nm. The hydrophobicity was calculated from the linear regression of initial slope of FI against protein concentration (mg/mL).

### 2.11. Water Holding Capacity (WHC)

HSPP (1 g) was weighed into a preweighed 15 mL centrifuge tubes [18]. For each sample, 10 mL of water was added and dispersed using a Vortex mixer for 2 min. The dispersion was allowed to stand at room temperature for 30 min, then centrifuged at 3000 ×g for 20 min at room temperature. The supernatant was decanted and the centrifuge tube containing sediment was weighed. The WHC (grams of water per gram of protein) was calculated using

(3) $$\begin{array}{c} WHC=\frac{\left(W_{2}-W_{1}\right)}{W_{0}}, \end{array}$$

where *W*_0_ is the weight of the dry sample (g), *W*_1_ is the weight of the tube plus the dry sample (g) and *W*_2_ is the weight of the tube plus the sediment (g).

### 2.12. Emulsion Properties Measurement

An oil-in-water (O/W) emulsion of HSPP was prepared by adding 8 mL of the soluble protein solution (1 mg/mL) to 2 mL of soybean oil and homogenizing it in a homogenizer (Fluko, Shanghai, China) at 20,000 rpm for 1 min [19]. The absorbance of the emulsion at 500 nm was recorded immediately (*A*_0_) and after 10 min (*A*_10_) using a spectrophotometer (SHJH Co. Ltd., Shanghai, China). The emulsifying activity index (EAI) and emulsion stability index (ESI) were determined using

(4) EAI  m2/g=2×2.303×A0×DFc×φ×1−θ×10,000ESI  min=A0A0−A10×10,

where *A*_0_ and *A*_10_ are the emulsion absorbance at 0 and 10 min; DF is dilution factor; *θ* is the fraction of oil used to form the emulsion (0.25); *φ* is the optical path (0.01 m); and *c* is protein concentration in sample solution (g/ml).

### 2.13. Foaming Properties

Foaming properties including foaming capacity (FC) and foam stability (FS) were determined using the method of Fernández-Quintela et al. with minor modifications [20]. Aliquots (10 mL) of sample solutions (1%, w/v) at pH 7.0 in measuring cylinder (25 mL) were homogenized with an FJ-200 high-speed homogenizer (Shanghai Co., China) at 10,000 rpm for 2 min. FC was calculated as the percent increase in volume of the protein dispersion upon mixing, while FS was estimated as the percentage of foam remaining after 30 min.

### 2.14. Statistical Analysis

All the experiments were conducted in triplicate and results were presented as mean ± standard deviation. Statistical analysis was performed on the obtained results using one-way analysis of variance (ANOVA) analysis and Turkey's test at *p* < 0.05 using SPSS (version 17.0, SPSS Inc., Chicago, ILL, USA).

## 3. Results and Discussion

### 3.1. Hydrolysis Degree Analysis

**Figure 1:**
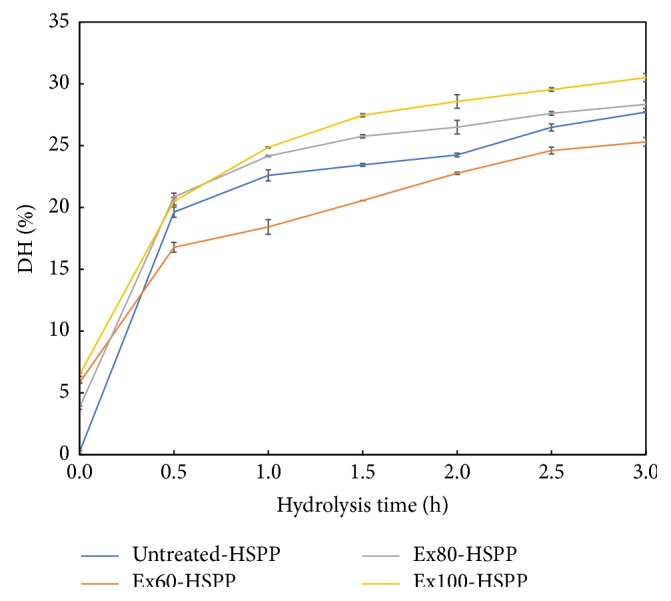
DH of hydrolyzed samples with/without extrusion.

The DH of HSPP is shown in Figure 1. The number of peptide bonds cleaved is quantified as DH [21]. Overall, the DH increased for all the test samples within the hydrolysis duration of 3 h. The biggest increase was observed at the first 0.5 h of hydrolysis. Extrusion at 60°C did not increase DH compared to the nonextruded control sample. Verbeek and Van Den Berg [22] demonstrated that the stabilizing force maintaining the tertiary and quaternary structures of the proteins was weakened by a combination of increased temperature and shear within the extruder. Simmons et al. [23] also reported that small protein aggregates were formed at 75°C during extrusion, whereas more rigid and dense protein aggregates were formed via disulfide interactions at high extrusion temperature over 80°C. Therefore, the significantly lowered DH of Ex60-HSPP may be due to the formed protein aggregates, which have a compact structure to impede hydrolysis. Klompong et al. [24] had similar observation that proteins with highly compacted structure were cleaved more slowly by enzymes. As temperature increased to 80 and 100, the protein aggregates tend to be broken, internal groups for enzyme hydrolysis [25]. Therefore, the formed rigid protein aggregates at the extrusion temperature of 80 and 100°C were further disrupted, resulting in a higher DH.

### 3.2. SDS-PAGE Analysis

**Figure 2:**
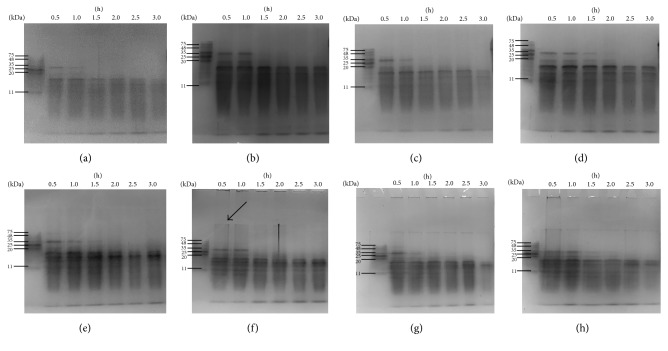
Reducing (a–d) and nonreducing (e–h) SDS-PAGE images of untreated and extruded HSPP samples: (a & e) nonextruded, (b & f) Ex60-HSPP, (c & g) Ex80-HSPP, and (d & h) Ex100-HSPP.

The nonreducing/reducing SDS-PAGE of extruded and nonextruded HSPP is shown in Figure 2. As expected, proteins were hydrolyzed to small peptides after hydrolysis. The HSPP profiles were slightly different under nonreducing and reducing conditions. Under reducing conditions, disulfide bridges in proteins are disrupted, whereas such interactions are preserved under nonreducing conditions. Therefore, the existence of disulfide bonds, which were suspected to be formed after extrusion, could be identified, as shown by the bands (pointed by an arrow) between the stacking gel and the separating gel, which represented the aggregated proteins. In comparison, no clear band was observed in the nonextruded sample, which further implied that the protein aggregations were formed during extrusion procedure.

### 3.3. High-Performance Size Exclusion Chromatogram

**Figure 3:**
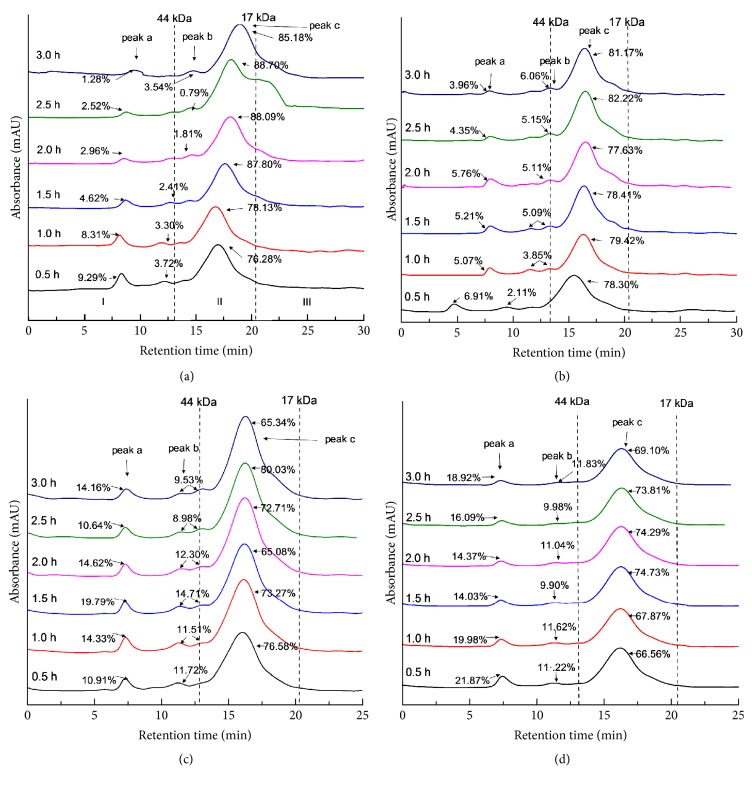
Molecular weight distributions of untreated and extruded HSPP samples: (a) nonextruded, (b) Ex60-HSPP, (c) Ex80-HSPP, and (d) Ex100-HSPP.

The molecular weight (MW) profiles of HSPP were measured by high-performance size exclusion chromatogram with the intention of understanding the dependence of functional properties of HSPP on molecular weights. As shown in Figures 3(a)–3(d), the MW distribution can be divided into three regions: (1) region I with MW above 44 kDa, (2) region II with MW between 17 and 44 kDa, and (3) region III with MW below 17 kDa.  Figure 3(a) shows that peak was minimized with the hydrolysis duration increase from 0.5 to 3.0 h, which was attributed to the formation of peptides with small MW due to enzymatic hydrolysis. However, upon extrusion, peaks (a and b) were enlarged and the size of peak (c) decreased. The aforementioned analysis of Section 3.1 implied that the extrusion cleaved the protein structure, exposing the hidden groups of protein to external environment. Sui et al. [13] found that the exposed anionic molecules within soy protein have emerged concomitantly with protein aggregation process. Therefore, it is assumed that the protein could form aggregates via intermolecular interactions during extrusion, resulting in the increase of MW. Similar results were reported by Nor Afizah and Rizvi [26] who suggested that protein aggregates were formed during extrusion. Although there was no significant difference in MW profile among the extruded samples, the functionalities may be significantly different.

### 3.4. Sulphydryl and Disulphide Bonds

**Figure 4:**
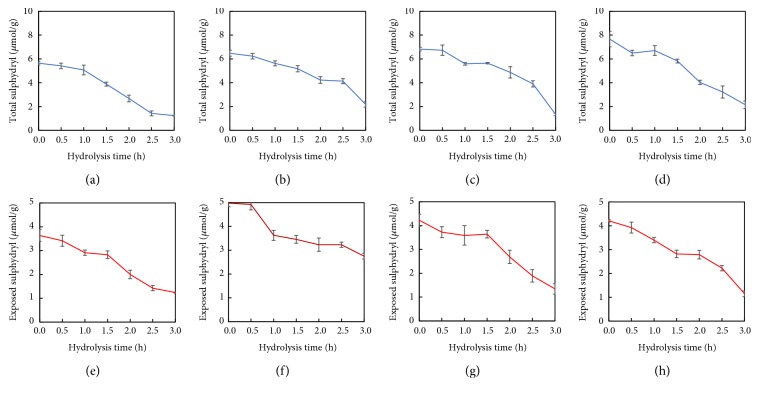
Total sulphydryl of (a) nonextruded, (b) Ex60-HSPP, (c) Ex80-HSPP, and (d) Ex100-HSPP. Exposed sulphydryl of (e) nonextruded, (f) Ex60-HSPP, (g) Ex80-HSPP, and (h) Ex100-HSPP.

**Figure 5:**
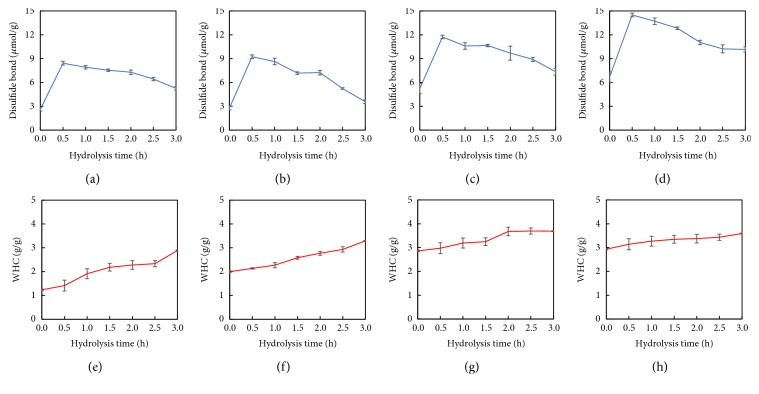
Disulfide bond of (a) nonextruded, (b) Ex60-HSPP, (c) Ex80-HSPP, and (d) Ex100-HSPP. WHC of (e) nonextruded, (f) Ex60-HSPP, (g) Ex80-HSPP, and (h) Ex100-HSPP.

Sulphydryl and disulphide bond plays an important role in the formation of relatively rigid structures and significantly influences the functional properties of proteins [27]. Figures 4 and 5(a)–5(d) show the total/exposed sulphydryl and disulphide bonds of HSPP. The exposed sulphydryls of HSPP were increased slightly relative to the nonextruded samples, suggesting that extrusion treatment unfolded the HSPP. It was found that hydrolysis led to a decrease of the exposed/total sulphydryl groups, with a sharp increase of disulphide bonds in HSPP relative to HSPP at 0 h. As reported by Creusot and Gruppen [28], hydrolysis can expose sulphydryl groups and promote further interaction between these active groups to form aggregates as seen in this work. With prolonging hydrolysis time, total/exposed sulphydryl and disulphide bond contents of HSPP decreased further, which might be due a breaking of the aggregate network by enzymatic hydrolysis. The results were similar to that for peanut protein and corn glutelin reported by Zhao et al. and Zheng et al. [29, 30]. At comparable hydrolysis times, Ex100-HSPP had a higher disulfide bond content than other samples. The disulfide bond content and the surface hydrophobicity were positively correlated with the emulsification indices of the peanut proteins/peptides. This suggested that Ex100-HSPP would have a better emulsion property.

### 3.5. WHC

All the extruded samples showed much higher WHC than that of nonextruded sample before hydrolysis (Figures 5(e)–5(h)). A similar tendency was observed by Alonso et al. [31] that extrusion resulted in an increase in the WHC of kidney beans or peas protein. Results indicated that the hydrolysis caused significant increases in the WHC of all the samples with or without extrusion. The current results are consistent with de Oliveira et al. who found that increasing DH of soy protein is accompanied by an increase in WHC [32]. The increased WHC was attributed to the fact that hydrolysis with Protex preserved more hydrophilic amino acid residues and reduced the hydrophobic sites, which increased the water retention of HSPP [33].

### 3.6. Solubility Measurement and Surface Hydrophobicity

**Figure 6:**
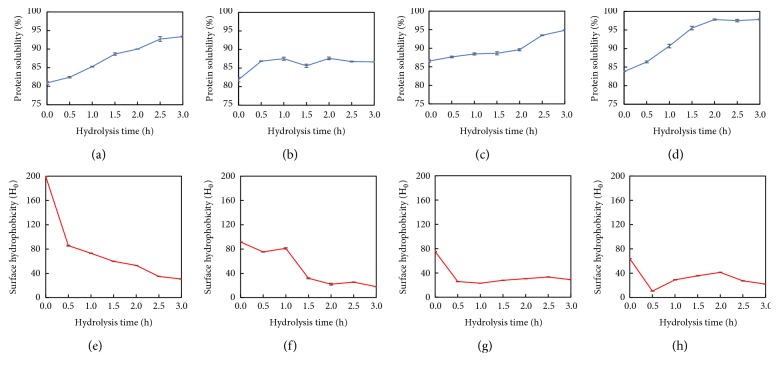
Protein solubility behavior of (a) nonextruded, (b) Ex60-HSPP, (c) Ex80-HSPP, and (d) Ex100-HSPP. Surface hydrophobicity behavior of (e) nonextruded, (f) Ex60-HSPP, (g) Ex80-HSPP, and (h) Ex100-HSPP.

The protein solubility is shown in Figures 6(a)–6(d). Overall, all the extruded samples showed higher initial solubilities at the hydrolysis time of 0 h than that of samples without extrusion. With increasing the hydrolysis time, the solubilities of extruded and nonextruded samples increased at different rates. Similar positive correlation was also observed between the extrusion temperature and solubility, in which the Ex100-HSPP showed the highest solubility. The peptides after hydrolysis of protein were able to form stronger hydrogen bonds with water and were more soluble in aqueous solutions [34, 35]. The formation of small peptides was probably the main reason for the increased solubility.

The surface hydrophobicity of proteins is one of the main structural characteristics used to evaluate changes in protein conformation [36]. Results indicated that the hydrolysis caused significant decreases in the hydrophobicity of all the samples with or without extrusion (Figures 6(e)–6(h)). The possible reason for the decreased hydrophobicity after hydrolysis is the increased enzymatic accessibility of HSPP, thereby causing the breakdown of hydrophobic groups during hydrolysis. Besides, the decreased hydrophobicity of HSPP might further be attributed to the formation of protein aggregates. Ryan et al. [37] found that hydrolyzed proteins were compared to nonhydrolyzed protein, in forming aggregates. It was also noted that all the extruded samples showed much lower hydrophobicity than that of nonextruded sample before hydrolysis. With an increase in the temperature of extrusion, the hydrophobicity decreased with Ex100-HSPP exhibiting the lowest initial hydrophobicity. A similar trend was also reported by Jung et al. [38] that extrusion treatment resulted in a decrease in the hydrophobicity of soy protein. This may be due to the extrusion-induced conformational changes of soy protein with a greater flexibility and more susceptible to enzymatic attack [39].

### 3.7. Emulsion Properties

**Figure 7:**
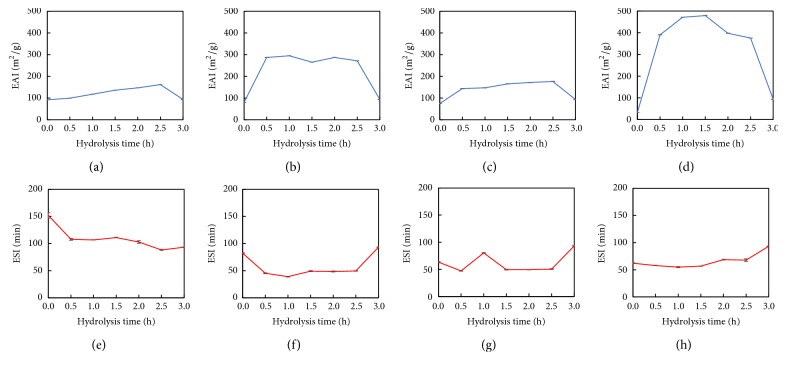
EAI and ESI analysis of (a & e) nonextruded, (b & f) Ex60-HSPP, (c & g) Ex80-HSPP, and (d & h) Ex100-HSPP.

The EAI is known to evaluate the effectiveness of proteins to preventing flocculation and coalescence in an emulsion. However, the EAI cannot descript the ability of proteins in maintaining a stable emulsion over periods, and thus the ESI was further adopted to fulfill this aim. As shown in Figures 7(a)–7(d), all the extruded samples showed higher EAI level than nonextruded sample. The increased EAI of Ex60-HSPP was attributed to the extrusion process, which made proteins more sensitive to enzymatic hydrolysis. Interestingly, the EAI did not increase continuously with an increase in the extrusion temperature to 80°C. The thermal denaturation temperatures of *β*-conglycinin and glycinin were reported to be 65–75 and 85–95°C [40]. Therefore, the reduced EAI of Ex80-HSPP was suspected to be the thermal-induced denaturation of glycinin. The EAI of Ex100-HSPP was much higher than all others, although both *β*-conglycinin and glycinin were thermally denatured at 100°C. This observation may seem to conflict with the above conclusion; however the higher solubility of Ex100-HSPP could explain the problem, since proteins with high solubilities can rapidly diffuse and adsorb at the interface of droplets to form a stable emulsion [40]. It reached a descriptive rather than prescriptive conclusion that the thermal-induced decrease in EAI of HSPP at 100°C was compensated by its high solubility. However, the EAI sharply decreased for all samples after 3 h of hydrolysis. Kristinsson and Rasco [41] reported a decreased EAI after enzymatic hydrolysis of 2 h, which may be due to the decreased protein peptide chain length. Mokni Ghribi et al. [42] similarly found that the hydrolysis process reduced the ability of smaller peptides to interact at the interface of droplets, causing the decreased EAI. Therefore, the decreased EAI after hydrolysis of 3 h was concluded to be due to the excessive hydrolysis.

As shown in Figures 7(e)–7(h), all the extruded samples showed larger decrease in ESI than the nonextruded sample. This indicated that the extruded proteins had weaker abilities to maintain a stable emulsion during storage, regardless their great effectiveness in forming emulsions, which was reflected by their higher EAI. Smaller peptides are known for their lower efficiency in stabilizing emulsions because they may not readily agglomerate to produce a fat globule membrane due to the charge repulsions and surface hydrophobicity [43, 44]. Thereafter, the smaller peptides formed during extrusion were considered as the major reason for the decreased ESI.

### 3.8. Foaming Properties

**Figure 8:**
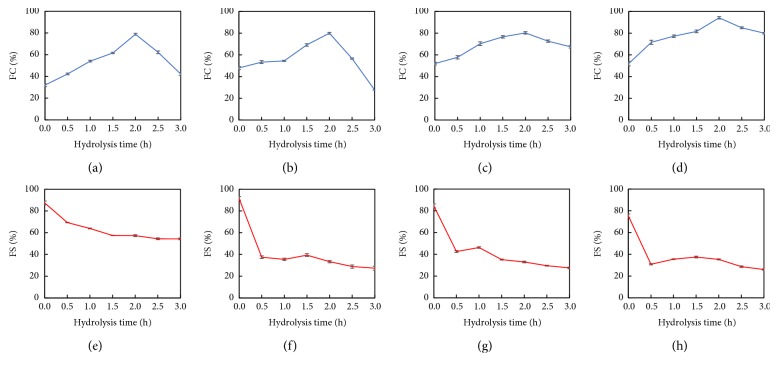
FC and FS analysis of (a & e) nonextruded, (b & f) Ex60-HSPP, (c & g) Ex80-HSPP, and (d & h) Ex100-HSPP.

As DH increased, the foaming capacity of HSPP increased (0–2.0 h) then decreased (2.0–3.0 h) as shown in Figures 8(a)–8(d). This could be due to the production of amphiphilic peptides after hydrolysis. Their reduced molecular weight will make them more flexible, forming a stable interfacial layer and increasing the rate of diffusion to the interface, improving the foamability properties. The large peptides and aggregates may have an inhibitory effect on the foaming properties by steric hindrance at the interface of the foam [45]. The higher FC of the Ex100-HSPP is consistent with the observed higher solubility and suggested that greater interactions with the aqueous phase enhance the ability of the protein molecules to encapsulate air particles. Interactions with the hydrophilic aqueous phase will enhance protein unfolding and hence result in better foam forming ability [46].

As shown in Figures 8(e)–8(h), enzymatic hydrolysis led to significant decreases in FS. Thus, the decreased chain length of peptides as a result of enzymatic hydrolysis may mainly account for the decrease in FS as with emulsion stability. The result are consistent with Wouters et al. [47]. The higher FS properties of nonextruded HSPP may be attributed to higher surface hydrophobicity, which enhance strong protein-protein interactions (as evident in Figure 5) and formation of a strong interfacial membrane at the air-water interface.

## 4. Conclusion

The hybrid extrusion-hydrolysis approach exhibited significant influence on the conformational and functional properties of soybean protein. Results showed that proteins formed more compacted aggregates at extrusion temperatures of 60°C than that formed at 80 and 100°C, which was indicated by the DH analysis. During extrusion, proteins might have interacted via disulfide bonds, forming aggregates with increased MW. With the introduction of enzymatic hydrolysis, the WHC and solubility of proteins increased and the hydrophobicity of proteins decreased. Extruded proteins showed higher EAI and FC than that of nonextruded proteins. However, excessive enzymatic hydrolysis reduced the EAI and FS due to the decreased protein peptide chain length.

## Data Availability

The data used to support the findings of this study are available from the corresponding author upon request.
